# Enhanced electrocatalytic activity of Pt-SnO_2_ nanoparticles supported on natural bentonite-functionalized reduced graphene oxide-extracted chitosan from shrimp wastes for methanol electro-oxidation

**DOI:** 10.1038/s41598-023-30705-w

**Published:** 2023-03-03

**Authors:** Ahmad Aryafar, Mehri-Saddat Ekrami-Kakhki, Atena Naeimi

**Affiliations:** 1 Department of Mining, Faculty of Engineering, University of Birjand, Birjand, Iran; 2 Central Research Laboratory, Esfarayen University of Technology, Esfarayen, North Khorasan Iran; 3 Department of Chemistry, Faculty of Science, University of Jiroft, Jiroft, Iran

**Keywords:** Electrochemistry, Nanoscale materials

## Abstract

In this work, tin (IV) oxide (SnO_2_) nanoparticles were synthesized based on *Amaranthus spinosus* plant. The produced graphene oxide by a modified Hummers’ method was functionalized with melamine (mRGO) and used accompanied by natural bentonite (Bnt) and extracted chitosan from shrimp wastes to prepare Bnt-mRGO-CH. This was utilized as novel support for anchoring Pt and SnO_2_ nanoparticles to prepare the novel Pt-SnO_2_/Bnt-mRGO-CH catalyst. The crystalline structure, morphology and uniform dispersion of nanoparticles in the prepared catalyst were determined by TEM images and XRD technique. The electrocatalytic performance of the Pt-SnO_2_/Bnt-mRGO-CH catalyst was evaluated for methanol electro-oxidation through electrochemical investigations including cyclic voltammetry, electrochemical impedance spectroscopy, and chronoamperometry techniques. Pt-SnO_2_/Bnt-mRGO-CH showed enhanced catalytic activity compared to Pt/Bnt-mRGO-CH and Pt/Bnt-CH catalysts considering its higher electrochemically active surface area, higher mass activity, and better stability for methanol oxidation. SnO_2_/Bnt-mRGO and Bnt-mRGO nanocomposites were also synthesized and did not show any significant activity for methanol oxidation. The results showed that Pt-SnO_2_/Bnt-mRGO-CH could be a promising catalyst as anode material in direct methanol fuel cells.

## Introduction

Today, with the development of urbanization and industrialization of cities and the growing demand for energy as well as the environmental pollution of fossil fuels, the attention of scientists has been drawn to the search for clean and efficient energy sources^1–3^. Among the various energy sources, direct methanol fuel cells (DMFCs) are of great attention due to their high energy density, easy storage and transportation of liquid fuel, low pollution emissions, and environmental compatibility^4–6^.

Platinum (Pt) is one of the efficient catalysts in direct methanol fuel cells, which has a good catalytic activity for methanol oxidation reaction (MOR). However, high cost, scarce natural reserve, strong affinity of Pt with carbon monoxide (CO) and poisoning with the adsorbed CO limit the use of Pt and its commercial application in fuel cells^7–9^. Therefore, one of the key challenges in DMFCs is to use new platinum-based catalysts with improved platinum catalytic activity and less carbon monoxide poisoning.

Several strategies have been applied to modify the usage of Pt catalyst in the fuel cells. The first strategy is to use other metals or metal oxides accompanied by Pt nanoparticles to enhance their catalytic performance for MOR^10–14^. Recent studies on metal oxides with great electrical conductivity property and an enormous number of oxygen vacancies have revealed that they might be a useful alternative for noble metals in fuel cells^15–17^. The use of metal oxides leads to improve alcohol oxidation and decreases the development of carbon monoxide (CO) through oxidizing carbon monoxide to carbon dioxide^18^. These features cause metal oxides to be applicable in direct alcohol fuel cells.

The second strategy is to increase the number of available active sites for MOR with the equal amounts of platinum loading. In order to increase the active surface and the presence of more platinum atoms on the catalysts' surface, the dispersion of nanoparticles should be improved and the size of platinum nanoparticles should be reduced. The use of appropriate supporting materials causes uniform dispersion of nanoparticles and prevents agglomeration of nanoparticles. Various support materials are used for dispersion of nanoparticles. Different carbon-based materials including carbon nanofibers, carbon nanotubes (CNTs), mesoporous carbon, graphene, Vulcan XC-72 carbon, and reduced graphene oxide (RGO) are used as supporting materials for Pt nanoparticles^19–23^. RGO is recognized as an appropriate supporting material due to its chemical stability, high charge mobility, great specific surface area, and excellent electronic conductivity One of the developing procedures to modify the reduced graphene oxide is functionalization of its surface which improves its stability and activity for nanoparticles^24, 25^.

Various polymers have been investigated as catalysts’ support in the fuel cells such as polyaniline, nafion, polypyrrole, polyvinyl alcohol, and chitosan^26–29^. Chitosan (CH) is a cheap, nontoxicity, renewable, numerous, and decomposable biopolymer with a robust dependency on metals that is formatted via the deacetylation of chitin^30, 31^. Chitosan has the amino (NH_2_) and OH functional groups in its structure, which cause this polymer to be a descent polymer electrolyte and an excellent catalysts’ support. The amino group in the structure of chitosan is easily protonated to produce NH_3_^+^ in acidic and neutral solutions^5^. Chitosan solution prepared through dissolving chitosan in 1% acetic acid aqueous solution and created an electrostatic attraction between PtCl_6_^2−^ and NH_3_^+^ with opposite charges.

In this work, RGO is functionalized with melamine to produce mRGO. Melamine (1,3,5-triazine-2,4,6-triamine) is a 6 nitrogen (N)-containing organic compounds with the chemical formula C_3_H_6_N_6_^32^. Bentonite (Bnt) is also utilized as catalyst support accompanied by mRGO and CH because its surface and special structure are very appropriate for transition metals. This clay is a nontoxic, freely available, inexpensive, and abundant natural mineral^33^.

In this work, for the first time, we report the utilization of Bnt-mRGO-CH as new catalyst support for direct alcohol fuel cells (DAFCs). The melamine and chitosan polymer are miscible in each other. The conductivity of the polymer would be increased due to the creation of robust hydrogen bonding among the amino groups of melamine and the hydroxyl groups of chitosan. These two would help the improved dispersion of Pt nanoparticles through the creation of the robust electrostatic attractions among the positively charged functional groups in their structure with the negatively charged PtCl_6_^2−^. Also, the catalysts’ thin layer can have better adherents in the presence of chitosan polymer on the surface of the working electrode. SnO_2_ nanoparticles were utilized with Pt nanoparticles to improve their catalytic performance for MOR. The catalytic performance of Pt-SnO_2_/Bnt-mRGO-CH nanocatalyst has been studied for methanol electro-oxidation in fuel cells through various electrochemical techniques including chronoamperometry, cyclic voltammetry, and electrochemical impedance spectroscopy (EIS).

## Experimental sections

### Materials

Bentonite and *Amaranthus spinosus* were collected from Birjand, Iran. 3-aminopropyl triethoxy silane, ethanol, and SnCl_2_.2H_2_O were bought from Merck. Chitosan was extracted from shrimp wastes. Powdered graphite (99.5%), nitric acid, sulfuric acid (H_2_SO_4_ 98%), hydrogen peroxide (30%), potassium permanganate (99%), hydrochloric acid (37%), sodium borohydride (96%), melamine, acetic acid (glacial, 100%), hexachloroplatinic acid, methanol (CH_3_OH 99.2%) were purchased from Merck.

### Synthesis of aminated bentonite

3 gr of bentonite was sonicated in 30 ml of ethanol. Then 5 mmol of 3-aminopropyl triethoxy silane was added and stirred within 24 h. The obtained aminated bentonite was filtered and washed with ethanol for three times.

### Synthesis of graphene oxide (GO)

Graphene oxide was obtained by a modified Hummers’ method from powdered graphite^34^. Briefly, sulfuric acid and nitric acids with the volume ratio of 3:1 and 2.5 g graphite powder were mixed by stirring for 24 h. the excess acids were removed by centrifuge. After drying, 25 ml acetone was added to the dried mixture using an ultrasonic bath for 30 min. After drying the mixture, 115 ml sulfuric was slowly added to the beaker under magnetic stirring. Afterwards, 15 g potassium permanganate was slowly added to the suspension in an ice bath. Following by the slowly addition of deionized water, the suspension was treated with 50 ml hydrogen peroxide. The product was centrifuged with hydrochloric acid 5% and washed with deionized water. The dried product was utilized as GO.

### Synthesis of melamine-RGO (mRGO)

To prepare melamine-RGO, 2 mg of graphene oxide was first mixed with 100 ml of deionized water and sonicated for 1 h. 10 mg of melamine was dissolved separately in 20 ml of deionized water and added to the first suspension and kept sonicating for another 1 h. Then, 30 μl of formaldehyde was poured into the mixture. While magnetic stirring, the NaOH solution was added drop by drop until the pH of the solution reached 9. The balloon lid was then closed and placed in a 70 °C water bath for 3 h. After that, 5–6 drops of APS liquid were added and the mixture was placed in a 70 °C water bath for another 3 h. After centrifuging, washing and drying the product, melamine reduced graphene oxide was prepared.

### Synthesis of bentonite-mRGO (Bnt-mRGO) nanocomposite

To prepare Bnt-mRGO nanocomposite, 3 gr of the aminated bentonite and 3 gr of mRGO were dispersed in 50 ml of water and refluxed within 5 h at 80 °C.

### Biosynthesis of SnO_2_ nanoparticles

The aqueous extract of *Amaranthus spinosus* (100 ml) was added dropwise to SnCl_2_.2H_2_O (25 ml, 0.05 M) with constant stirring at room temperature for 30 min. After 5 min, the solution color was changed from white to brown indicating the formation of the desired nanoparticles. Stirring of the solution was continued for another 30 min at 75 °C and after that, the obtained solution was cooled and centrifuged at 5000 rpm. The resultant precipitates were washed several times with double distilled water and dried at room temperature. The obtained nanoparticles were grounded using an agate mortar and then placed inside the furnace at 550 °C for 3 h. The SnO_2_ nanoparticles were obtained as a beige powder.

### Synthesis of SnO_2_/Bnt-mRGO nanocomposite

To prepare SnO_2_/Bnt-mRGO nanocomposite, 2 gr of Bnt-mRGO and 2 gr of SnO_2_ nanoparticles were added to 30 ml of deionized water and refluxed within 6 h. The obtained nanocomposite was washed three times with double distilled water.

### Synthesis of Pt-based catalysts

Pt-SnO_2_/Bnt-mRGO-CH catalyst was synthesized with a simple procedure as follows: 1 mg Bnt-mRGO and 2 mg of SnO_2_ nanoparticles were uniformly dispersed in deionized water and chitosan (Volumetric ratio: 17.5/2.5) after sonication for 1 h. 25 µl H_2_PtCl_6_ (1 M) was poured into the mixture and vigorously stirred for 1 h. Afterwards, 50 µl aqueous solution of NaBH_4_ (3 M) was quickly injected to the suspension, while kept stirring for another 24 h. The resulting black suspension was centrifuged and washed several times with deionized water for purification. Finally, the product was dried at 50 °C for 12 h and the Pt-SnO_2_/Bnt-mRGO-CH catalyst was obtained. Pt/Bnt-mRGO-CH catalysts were synthesized by the identical procedure without using the tin oxide nanoparticles. Bnt-CH support was subjected to the same procedure to obtain Pt/Bnt-CH catalyst.

### Physical characterization

The synthesized GO was characterized by X-ray diffraction instrument (XRD, Philips PC-APD apparatus equipped with a graphite monochromatic CuKa radiation source). The morphological and structural information of the catalysts were obtained by XRD and transmission electron microscope (TEM, Philips CM120) images. The prepared support materials were analyzed by Fourier-transform infrared (FT-IR) spectrophotometer (Brucker, TENSOR 27). The amount of Pt nanoparticles, loaded in the catalysts, was determined by inductively coupled plasma optical emission spectroscopy (ICP-OES, Shimadzu ICPE-9800).

### Electrochemical measurements

A uniform catalyst ink was obtained by dispersing 2 mg of catalyst powder in 1 ml of chitosan solution, followed by ultrasonic treatment for 15 min. Then, a uniform thin catalyst film was obtained by dropping 5 μl of the catalyst ink onto the surface of glass carbon (GC) electrode with 2 mm diameter and drying for overnight.

A potentiostat/galvanostat Autolab apparatus (Nova software, model PGSTAT 302N, Metrohm, Netherlands) with a classical three-electrode cell was utilized to perform the electrochemical investigations of the catalysts. A saturated calomel electrode (SCE) was served as reference electrode. The platinum and GC electrodes were employed as auxiliary and working electrodes, respectively. The amount of Pt nanoparticles on the surface of the modified GC electrodes, assessed by ICP-OES, was determined to be 3.134 μg for the synthesized catalysts.

## Results and discussion

### Material characterization

**Figure 1:**
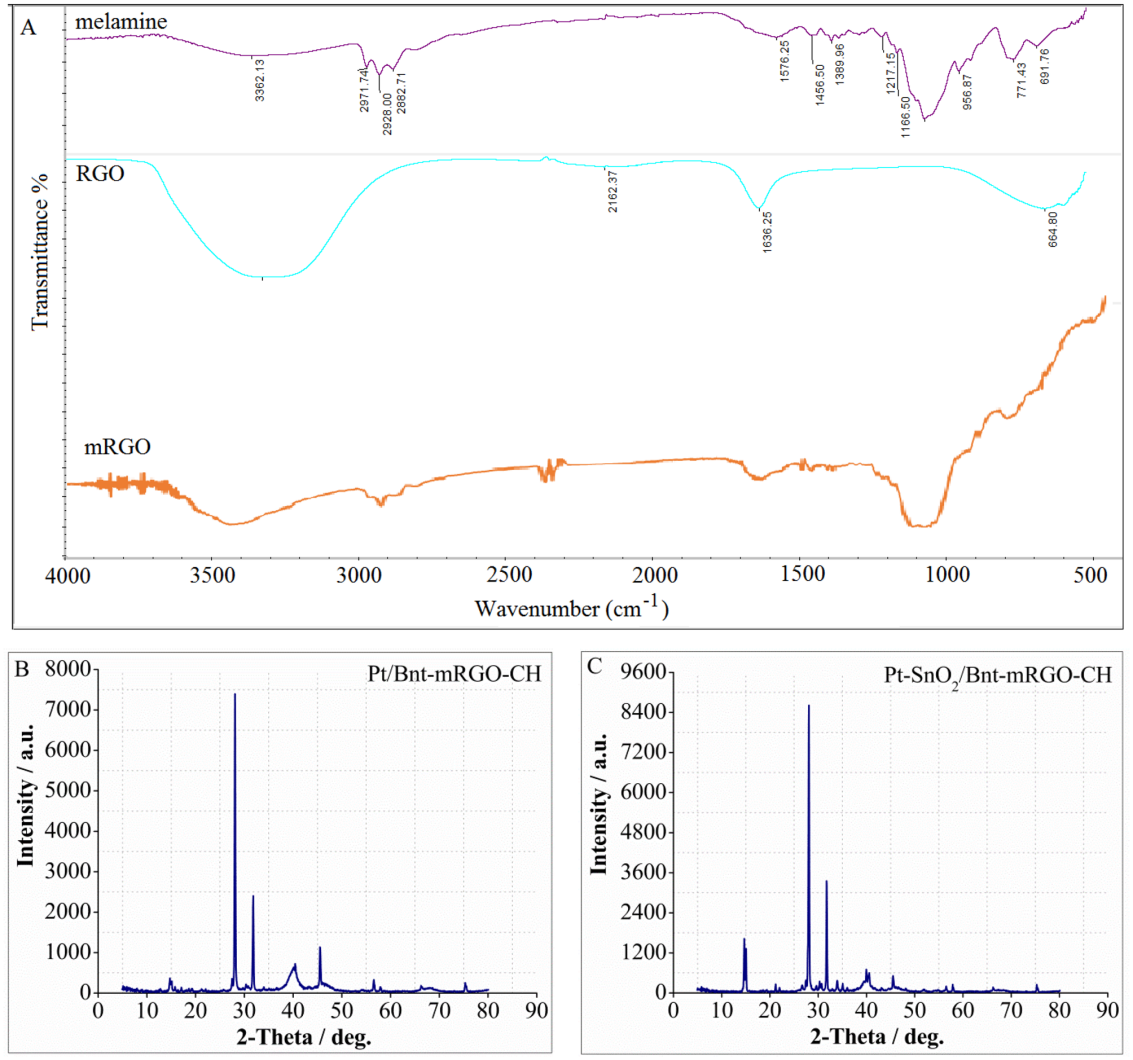
(**A**) FTIR spectra of melamine, RGO and mRGO. (**B**) XRD pattern of Pt/Bnt-mRGO-CH and (**C**) XRD pattern of Pt-SnO_2_/Bnt-mRGO-CH catalyst.

FT-IR spectroscopy is a power full technique to characterize the presence of different functional groups in chemical materials. FT-IR spectra of GO, melamine and mRGO are shown in Fig. 1A. In the FT-IR spectrum of melamine, the absorption peaks at 3362.13 cm^−1^ are assigned to –NH_2_ stretching vibration; whereas those at 1576 cm^−1^ and 956 cm^−1^ are assigned to the triazine ring. The stretching (N–)–C = O peak at 1666 cm^−1^ may overlap by ring C = N, ring C–N stretching (1554 cm^−1^) and carbonyl (C = O) for ketone at (1718 cm^−1^). The ring C–N bending (814 cm^−1^), stretching C–N (1144 cm^−1^), C–C (1479 cm^−1^), and stretching C–O (1402 cm^−1^) peaks show the successful functionalization process^35, 36^. In FT-IR spectrum of RGO, the deformation of water molecules at 1636 cm^−1^ was observed. Further the bands at 1586 cm^−1^ corresponds to C = C in-plane vibrations and 664 cm^−1^ is corresponding to –CH out-of-plane vibrations. A broad peak at 3400 cm^−1^ can be assigned to the vibrations of the adsorbed water molecules or may also contain skeletal vibrations corresponding to unoxidized graphitic domains. The spectrum does not contain any peaks associated with C–OH (~ 1340 cm^−1^), C = C (~ 1570 cm^−1^), and –COOH (~ 1710–1720 cm^−1^) groups. The C = C bonds missing indicates a strong oxidation to have taken place^37–39^.

XRD patterns of Pt/Bnt-mRGO-CH and Pt-SnO_2_/Bnt-mRGO-CH catalysts are shown in Fig. 1B and C, respectively. In XRD pattern of Pt/Bnt-mRGO-CH, the main constituents of bentonite are montmorillonite, quartz and feldspar which their diffraction patterns are completely shown in Fig. 1B. The diffraction peak, located at 2θ = 35.92° corresponds to (006) plane of montmorillonite^40^. The XRD peaks positioned at 2θ = 21.94°, 28.41° and 35.92° are relevant to (101), (111) and (102) planes of cristobalite, respectively. The presence of quartz is also indicated by a peak at 2θ = 26.53° (011). Furthermore, the diffraction peaks of Pt nanoparticles are observed at 39.84, 46.74 and 67.38 which are corresponded to the (111), (200) and (220) planes of Pt, respectively^5^. RGO usually shows a very broad weak diffraction peak at 2θ = 24° (002) due to the lack of crystallinity of graphene. In the XRD pattern of Pt/Bnt-mRGO-CH, (002) usually does not appear because the diffraction peaks of bentonite nanocrystals are much stronger than the (002) of RGO^41^. In XRD pattern of Pt-SnO_2_/Bnt-mRGO-CH, the observed peaks at 2θ = 26.48, 33.52, 37.80, and 51.64 correspond to the (1 1 0), (1 0 1), (2 0 0), and (2 1 1) planes of SnO_2_ nanoparticles, respectively. The XRD pattern of the synthesized nanoparticles exactly matches with the tetragonal rutile structure of SnO_2_ nanoparticles (JCPDS 77-0448)^42^.

**Figure 2:**
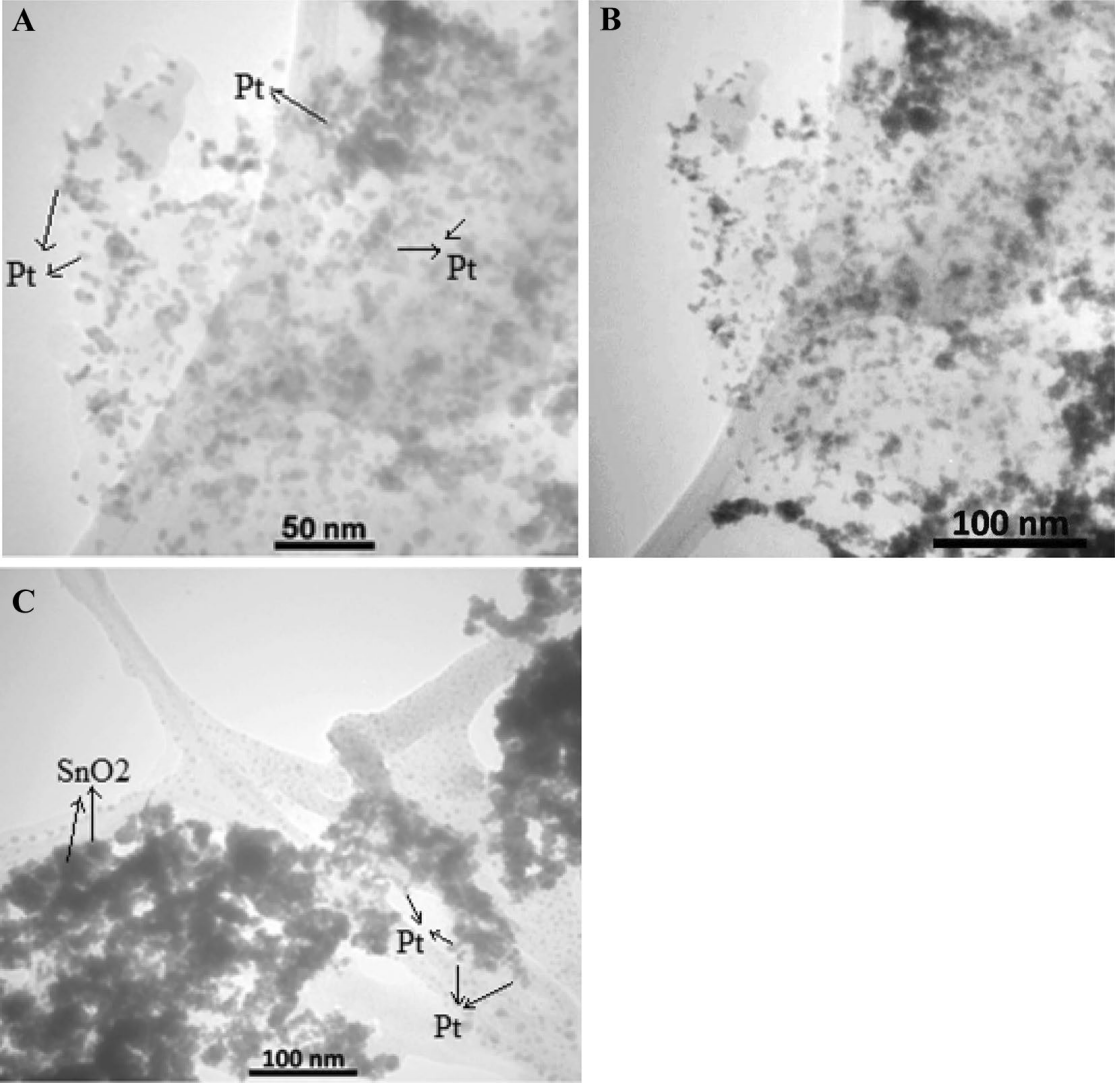
TEM images of (**A**), (**B**) Pt/Bnt-mRGO-CH and (**C**) Pt-SnO_2_/Bnt-mRGO-CH nanocatalysts.

TEM images of Pt/Bnt-mRGO-CH and Pt-SnO_2_/Bnt-mRGO-CH nanocatalysts are shown in Fig. 2A–C, respectively. The presence of platinum and tin oxide nanoparticles, dispersed in the Bnt-mRGO-CH substrate, is clearly observed. Platinum nanoparticles are uniformly dispersed in Bnt-mRGO-CH substrate and around tin oxide nanoparticles. The average size of the spherical platinum particles is about 5 nm and tin nanoparticles are about 40 nm.

### Electrocatalytic studies

#### Hydrogen adsorption and desorption

**Figure 3:**
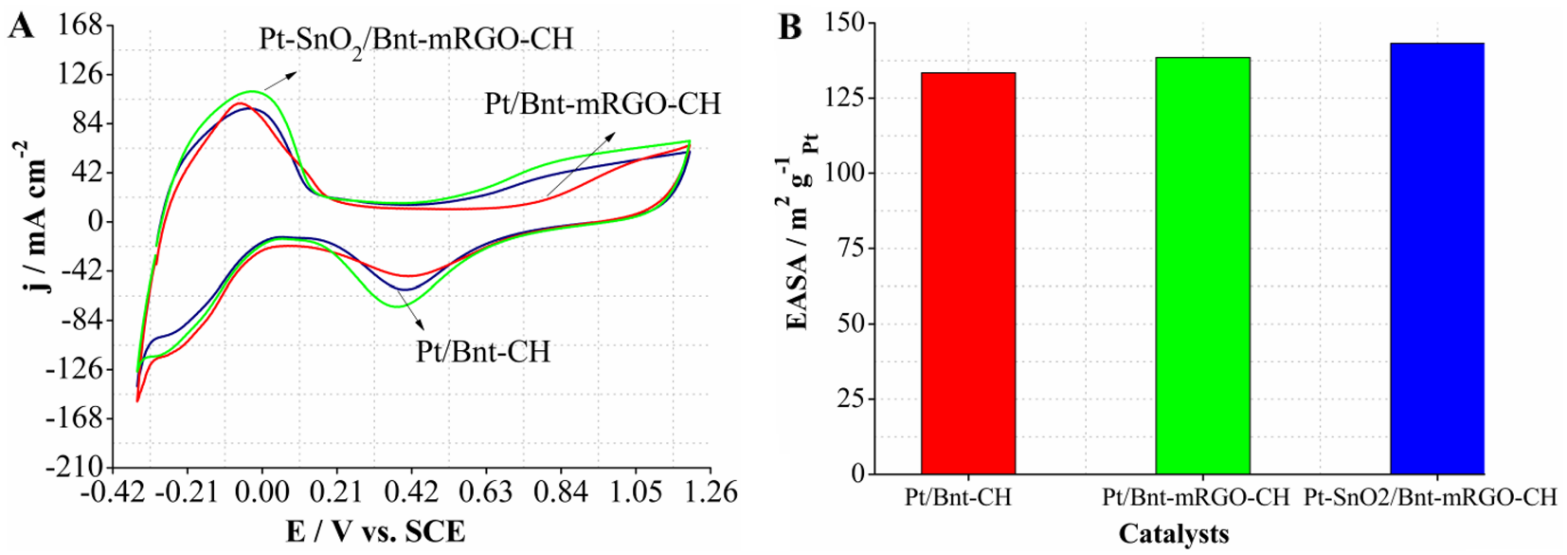
(**A**) Cyclic voltammograms and (**B**) EASA values of the catalysts in 0.5 M H_2_SO_4_.

To evaluate the number of effective catalytic sites of the Pt-based catalysts for MOR, electrochemically active surface area (EASA) of the synthesized catalysts was determined. For this purpose, the cyclic voltammetry (CV) curves of the catalysts were obtained in sulfuric acid solution (0.5 M) in the potential range from − 0.35 to 1.2 V and scan rate of 100 mV s^−1^ (Fig. 3A). All the synthesized catalysts showed two pronounced peaks in the potential range between − 300 mV to 100 mV which are attributed to the hydrogen desorption and absorption on the Pt surface. EASA was obtained by integrating the hydrogen desorption and adsorption regions according to the following equation^43^:

1 $$EASA= Q_H / (0.21 \times [Pt])$$

Q_H_ (mC cm^−2^) is the mean charge value passed during hydrogen adsorption and desorption, 0.21 is the constant value which is related to the charge, required for oxidizing one monolayer of hydrogen on the Pt nanoparticles’ surface. [Pt] (mg cm^−2^) is the amount of platinum, loaded on the surface of modified working electrode^25^. As calculated, the EASA value of Pt-SnO_2_/Bnt-mRGO-CH catalyst was 143.182 m^2^ g^−1^_Pt_, which was the maximal value relative to Pt/Bnt-mRGO-CH (138.502 m^2^ g^−1^_Pt_) and Pt/Bnt-CH (133.41 m^2^ g^−1^_Pt_) catalysts (Fig. 3B). With the same amount of Pt loading, the higher EASA value of Pt-SnO_2_/Bnt-mRGO-CH, compared to Pt/Bnt-mRGO-CH and Pt/Bnt-CH catalysts reveals its higher accessible active sites for methanol oxidation^44^. The higher EASA value of Pt-SnO_2_/Bnt-mRGO-CH, compared to the other two catalysts is probably due to the better dispersion of Pt nanoparticles in this catalyst. As shown in the TEM images of Pt-SnO_2_/Bnt-mRGO-CH, Pt nanoparticles are dispersed in the matrix of Bnt-mRGO-CH support and around SnO_2_ nanoparticles. In addition, the presence of hydroxyl-rich species produced from water dissociation on the surface of tin oxide nanoparticles facilitates the removal of hydrogen, adsorbed during the hydrogen oxidation reaction, and enhances the hydrogen oxidation reaction^45^. The dispersion of Pt nanoparticles in the synthesized Pt-based catalysts can be determined from the EASA values according to the following equation^46^:

2 $$D_(Pt )= EASA /(1/ M_Pt (N_A, 4\pi r_Pt^2)),$$

where, M_Pt_ and r_Pt_ are the relative molecular weight (195.08 g/mol) and the atomic ratio (0.139 nm) of Pt, respectively. N_A_ is related to the Avogadro number (6.02 × 10^23^). D_Pt_ values of Pt-SnO_2_/Bnt-mRGO-CH, Pt/Bnt-mRGO-CH, and Pt/Bnt-CH catalysts were obtained as 0.191, 0.185, and 0.178, respectively. As shown, the presence of mRGO accompanied by Bnt-CH and also the presence of SnO_2_ nanoparticles improve the dispersion of Pt nanoparticles on the electrode surface.

#### Electrochemical activity for methanol oxidation reaction (MOR)

**Figure 4:**
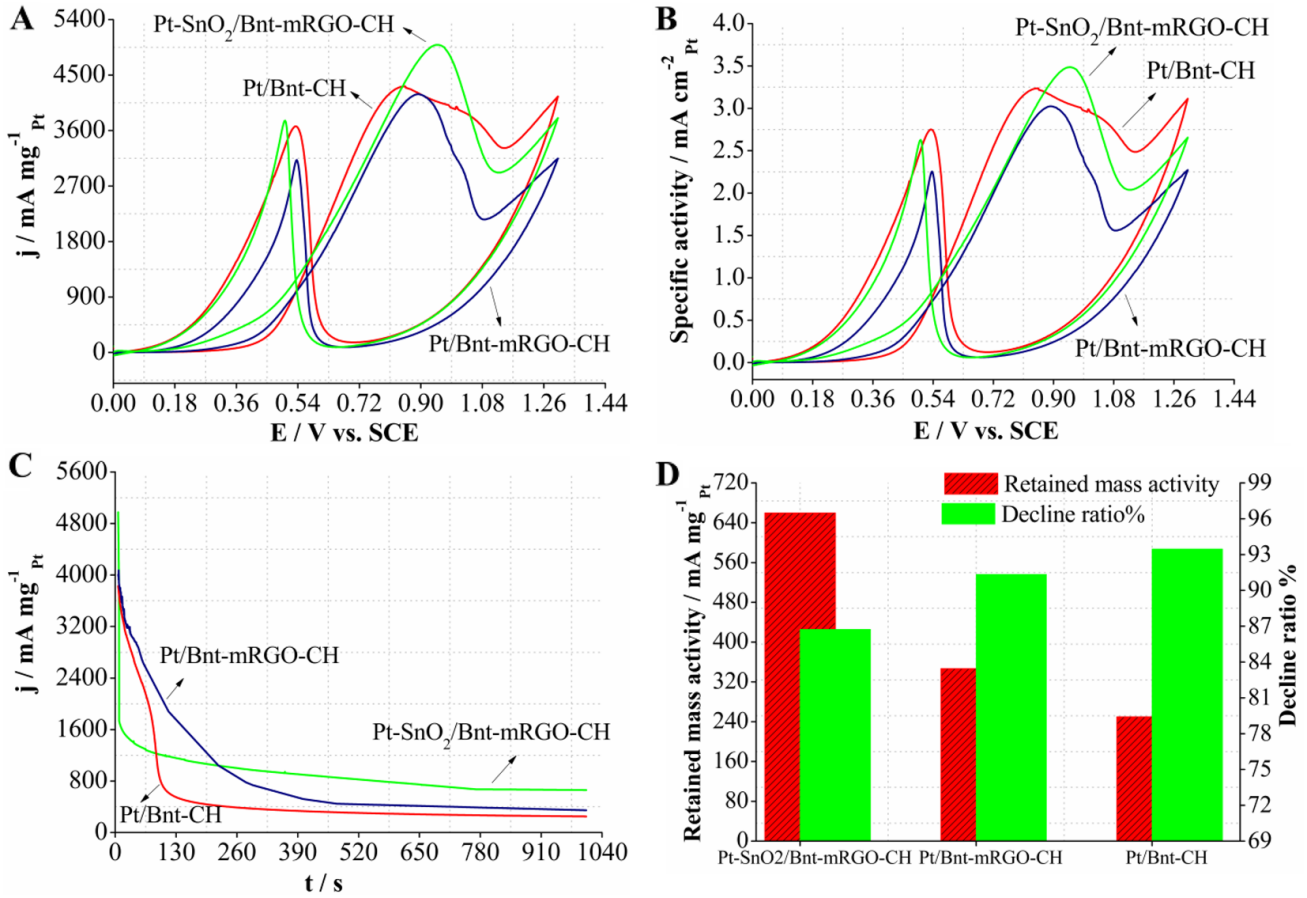
(**A**) The mass activity, (**B**) the specific activity and (**C**) the chronoamperometric curves of the catalysts in sulfuric acid 0.5 M and methanol 0.72 M. (**D**) the retained mass activity and activity decline ratio of the catalysts after 1000 s.

The evaluation of the electrochemical performance of Pt-SnO_2_/Bnt-mRGO-CH, Pt/Bnt-mRGO-CH and Pt/Bnt-CH nanocatalysts for MOR was conducted in an aqueous mixed solution of sulfuric acid (0.5 M) and methanol (0.72 M) (Fig. 4A,B). All the CV curves displayed two intense peaks for MOR. The first peak in the forward scan (j_f_) belongs to the methanol electro-oxidation and the second one in the backward scan (j_b_) is attributed to the oxidation of the accumulated intermediates, formed during the forward scan^47^.

Insights into catalytic activity of the synthesized catalysts for MOR are obtained by comparing their anodic peak current and potential, and onset potential for methanol electro-oxidation. The mass activity of the prepared catalysts was obtained from the current normalized by the mass of loaded platinum on the surface of modified working electrodes. As clearly shown in Fig. 4A, Pt-SnO_2_/Bnt-mRGO-CH has improved electrocatalytic activity, compared to Pt/Bnt-mRGO-CH and Pt/Bnt-CH catalysts for MOR.

The onset potential of MOR for Pt-SnO_2_/Bnt-mRGO-CH catalyst was at 0.18 V whereas, for Pt/Bnt-mRGO-CH and Pt/Bnt-CH catalysts were at 0.236 V and 0.322 V, respectively. The onset potential of MOR at the prepared catalysts is related to the breaking of carbon-hydrogen bonds and removal of the absorbed carbon monoxide species^48^. The more negative onset potential of Pt-SnO_2_/Bnt-mRGO-CH for methanol oxidation, compared to the other two catalysts is probably due to the presence of tin oxide nanoparticles, which facilitates the removal of adsorbed carbon monoxide from the catalyst surface^49^.

The normalized anodic peak mass activity of MOR on the surface of Pt-SnO_2_/Bnt-mRGO-CH catalyst was 4986.615 mA mg^−1^_Pt_, which was higher than that of Pt/Bnt-mRGO-CH (4189.107 mA mg^−1^_Pt_) and Pt/Bnt-CH (4316.67 mA mg^−1^_Pt_) catalysts. The mass activity ratio of the anodic peak in the forward scan to the backward scan (j_f_/j_b_) can be used to compare the catalytic performance of the synthesized catalysts for MOR. This ratio can be utilized to determine the tolerance of the prepared catalysts to carbon monoxide species, produced during methanol electro-oxidation. The larger values of j_f_/j_b_ indicate that the synthesized catalysts have better resistance to carbon monoxide poisoning^50^. The j_f_/j_b_ value for Pt-SnO_2_/Bnt-mRGO-CH catalyst was 1.33, while this ratio for Pt/Bnt-mRGO-CH and Pt/Bnt-CH catalysts was 1.31 and 1.17, respectively. The higher j_f_/j_b_ ratio for Pt-SnO_2_/Bnt-mRGO-CH catalyst indicates its enhanced catalytic performance for MOR, compared to the other two catalysts. In fact, incorporating SnO_2_ nanoparticles in bentonite-melamine graphene oxide-chitosan substrate and using them together with platinum nanoparticles increases the catalytic performance of platinum for MOR. This is related to the presence of SnO_2_ nanoparticles, which facilitate methanol electro-oxidation by producing more hydroxyl groups. The mechanism of methanol oxidation on the surface of Pt-SnO_2_ nanoparticles is considered as follows^16, 51^:

3 $$\mathrm{Pt}+{\mathrm{CH}}_{3}\mathrm{OH }\to \mathrm{Pt}-{\mathrm{CO}}_{\mathrm{ads}}+4\mathrm{e}+4{\mathrm{H}}^{+}$$

4 $$\mathrm{Pt }+ {\mathrm{H}}_{2}\mathrm{O }\to \mathrm{Pt}-{\mathrm{OH}}_{ads} + {\mathrm{H}}^{+} +\mathrm{ e}$$

5 $$\mathrm{Pt}-{(\mathrm{CO})}_{ads} +\mathrm{ Pt}-{\mathrm{OH}}_{ads} \to 2\mathrm{Pt }+\mathrm{ C}{\mathrm{O}}_{2} + {\mathrm{H}}^{+} +\mathrm{ e }$$

6 $$\mathrm{M}+ {\mathrm{H}}_{2}\mathrm{O}\to \mathrm{M}-{\mathrm{OH}}_{\mathrm{ads}}+\mathrm{e}+ {\mathrm{H}}^{+}$$

$$\mathrm{M}=\mathrm{S}n{O}_{2}$$

7 $$\mathrm{Pt}-\mathrm{CO}+\mathrm{M}-{\mathrm{OH}}_{\mathrm{ads}}\to {\mathrm{CO}}_{2}+\mathrm{e}+{\mathrm{H}}^{+}+\mathrm{Pt}+\mathrm{M}$$

At first, methanol is adsorbed and dehydrogenated on the Pt nanoparticles’ surface. Pt nanoparticles are poisoned by the adsorbed carbon monoxide (CO) species on their surface. Methanol oxidation is prevented on the surface of the poisoned Pt nanoparticles. In order to oxidize CO intermediates to CO_2_, the adsorbate should react with such oxygen-containing species as OH_ads_ and H_2_O in the aqueous solution. The presence of SnO_2_ nanoparticles, accompanied by Pt nanoparticles, can accelerate the production of hydroxyl species. These hydroxyl species can facilitate oxidation and removal of the adsorbed CO from the surface of Pt nanoparticles, and thus prevent poisoning of Pt-SnO_2_/Bnt-mRGO-CH catalyst with CO^52, 53^.

The specific activity, normalized to the EASA values, is also calculated to evaluate the intrinsic activity of the catalysts (Fig. 4B)^54^. As expected, the highest specific activity is observed on Pt-SnO_2_/Bnt-mRGO-CH (3.483 mA cm^−2^), which is higher than that of Pt/Bnt-mRGO-CH (3.024 mA cm^−2^) and Pt/Bnt-CH (3.235 mA cm^−2^) catalysts. This result confirmed the enhanced activity of Pt-SnO_2_/Bnt-mRGO-CH for MOR among all the examined catalysts.

Chitosan and melamine reduced graphene oxide have NH_2_ and OH functional groups in their structures. These functional groups are positively charged in an acidic environment. A more uniform dispersion of Pt nanoparticles and their better catalytic performance for MOR were obtained due to the electrostatic attraction between the positively charged functional groups and PtCl_6_^2-^ with negative charge^55^. In addition, the presence of chitosan causes better adhesion of the catalyst layer to the electrode surface. The large surface area, mechanical properties, high thermal stability and other unique properties of bentonite have led to its wide use in adsorbents and composites^56^. In green chemical processes, bentonite has a very high potential to absorb palladium and platinum on its surface and create a stable composite^57^.

The catalytic activity of the prepared catalysts was compared with that of other catalysts and the commercial Pt/C, mentioned in the literature^16, 18, 44, 51, 58–66^. As shown in Table 1, Pt-SnO_2_/Bnt-mRGO-CH has higher catalytic activity than other catalysts towards methanol oxidation according to its higher EASA and higher anodic mass activity. Higher mass activity of Pt-SnO_2_/Bnt-mRGO-CH catalyst, compared to that of the other mentioned catalysts, is probably due to the small size of nanoparticles, their homogeneous and uniform distribution on the Bnt-mRGO-CH support and the presence of SnO_2_ nanoparticles, accompanied by Pt nanoparticles for methanol oxidation^16, 60^.

**Table 1:** Electrochemical data of methanol electro-oxidation at various catalysts.

Catalysts	EASA (m^2^ g^−1^_Pt_)	E_f_ (V) vs. SCE	j_f_ (mA mg^−1^_Pt_)	Ref
Pt/PVA-CuO-Co_3_O_4_/CH	54.56	0.810	3010.868	^16^
Pt/PVA-CuO-Co_3_O_4_	35.89	0.744	1597.984	^16^
Pt/LP-TiO_2_/CFP	230.04	0.7	1182.8	^51^
PtPdCr/C	65	0.69	969	^58^
Pt-Pb HAc/C	10.53	0.98 RHE	1000	^59^
GCE/PBCB/Pt-Ru	302.8	0.69 Ag/AgCl	898.6	^60^
Pt-C/Fe_2_-CoP	117.34	0.7	1237	^44^
Pt-FeNi_2_P/C	90.47	0.641	1125	^61^
Pt/C-Au@CeO_2_-Pt	77.8	0.8	1267	^18^
Commercial Pt/C	45.6	0.7	616	^18^
Commercial Pt/C	74.62	0.7	470	^62^
Commercial Pt/C (20% Pt)	65.7	0.65	258.5	^63^
Pt NPs/C	22	0.65	153.8	^64^
Pt NWs/NL-CNS	115.9	0.7	1949.5	^64^
Pt/C (C:Vulcan carbon)	39.67	0.62	192.97	^65^
Pt/OMCS (ordered mesoporous carbon sphere)	73.5	0.9 vs. NHE	510	^66^
Pt/Bnt-CH	133.41	0.854	4316.67	This work
Pt/Bnt-mRGO-CH	138.502	0.891	4189.107	This work
Pt-SnO_2_/Bnt-mRGO-CH	143.182	0.942	4986.615	This work

The electrochemical stability of the synthesized catalysts was evaluated using chronoamperometric curves at 0.85 V for 1000 s in 0.5 M H_2_SO_4_ and 0.72 M methanol solutions (Fig. 4C). As can be seen, the activities of the prepared catalysts decrease rapidly in the initial stage due to the poisoning of platinum nanoparticles with the adsorbed chemical species such as carbon monoxide, formed during methanol electro-oxidation^67–69^.

The Pt-SnO_2_/Bnt-mRGO-CH catalyst has the highest initial mass activity and the lowest mass activity loss after 1000 s, compared to the other two catalysts. After 1000 s, Pt-SnO_2_/Bnt-mRGO-CH (659.637 mA mg^−1^_Pt_) showed the highest residual mass activity, compared to the Pt/Bnt-mRGO-CH (346.812 mA mg^−1^_Pt_) and Pt/Bnt-CH (249.932 mA mg^−1^_Pt_) catalysts (Fig. 4D). The chronoamperometric results showed that Pt-SnO_2_/Bnt-mRGO-CH catalyst has better stability and catalytic performance for methanol electro-oxidation, compared to the other two catalysts.

**Figure 5:**
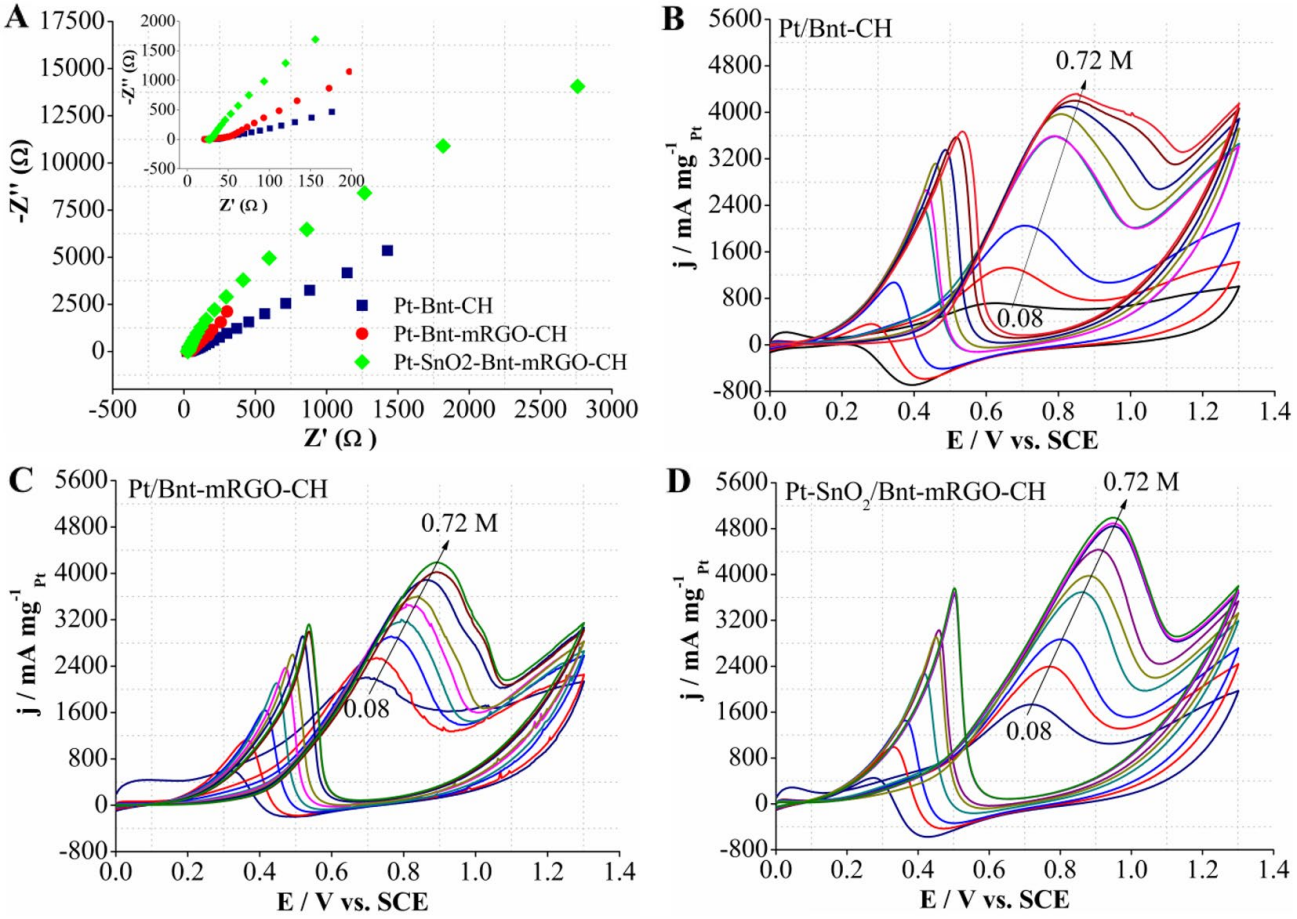
(**A**) Nyquist plots of the catalysts for MOR in 0.5 M H_2_SO_4_ and 0.72 M methanol, CV curves on (**B**) Pt/Bnt-CH, (**C**) Pt/Bnt-mRGO-CH and (**D**) Pt-SnO_2_/Bnt-mRGO-CH in 0.5 M H_2_SO_4_ and different concentration of methanol 0.08, 0.16, 0.24, 0.32, 0.41, 0.48, 0.56, 0.64 and 0.72 M.

Electrochemical impedance spectroscopy (EIS) is utilized to evaluate the electrode kinetics under MOR. As shown in Fig. 5A, all the synthesized catalysts exhibit the typical Nyquist plots. The Nyquist plots were obtained in the range of 1 × 10^4^ to 10^−2^ Hz at open circuit potential (OCP) in 0.5 M H_2_SO_4_ and 0.72 M methanol. All the Nyquist plots showed a small semicircle in high frequency region and a line in the low-frequency region^70^. The diameter of the Nyquist plots semicircle, in the high frequency range, is related to the charge-transfer resistance during MOR. The smaller semicircles’ diameter of Pt-SnO_2_/Bnt-mRGO-CH signifies its faster catalytic kinetic rate for MOR, compared to the other two catalysts.

The effects of different methanol concentrations on the anodic mass activity of methanol electro-oxidation at Pt-SnO_2_/Bnt-mRGO-CH, Pt/Bnt-mRGO-CH and Pt/Bnt-CH catalysts are represented in Fig. 5. As shown, the anodic mass activity of MOR at the prepared catalysts increases with the increase in methanol concentration up to 0.72 M. At higher methanol concentration, the anodic mass activity of MOR does not change with the increase of methanol concentration, and this is probably due to the saturation of the active sites on the surface of the electrodes^71^. In addition, with increase in methanol concentration, the anodic potential of methanol oxidation shifts to more positive potentials. This is probably due to the enhanced poisoning of platinum nanoparticles^72^. For Pt-SnO_2_/Bnt-mRGO-CH (Fig. 5D), with increasing the methanol concentration from 0.08 M to 0.72 M, the anodic mass activity of methanol oxidation increases from 1731.147 to 4986.615 mA mg^−1^_Pt_. While for Pt/Bnt-mRGO-CH (Fig. 5C) and Pt/Bnt-CH (Fig. 5B) catalysts with increasing the methanol concentration from 0.08 M to 0.72 M, the anodic mass activity of MOR increases from 2203.615 to 4189.107 mA mg^−1^_Pt_ and from 715.031 to 4316.67 mA mg^−1^_Pt_, respectively.

**Figure 6:**
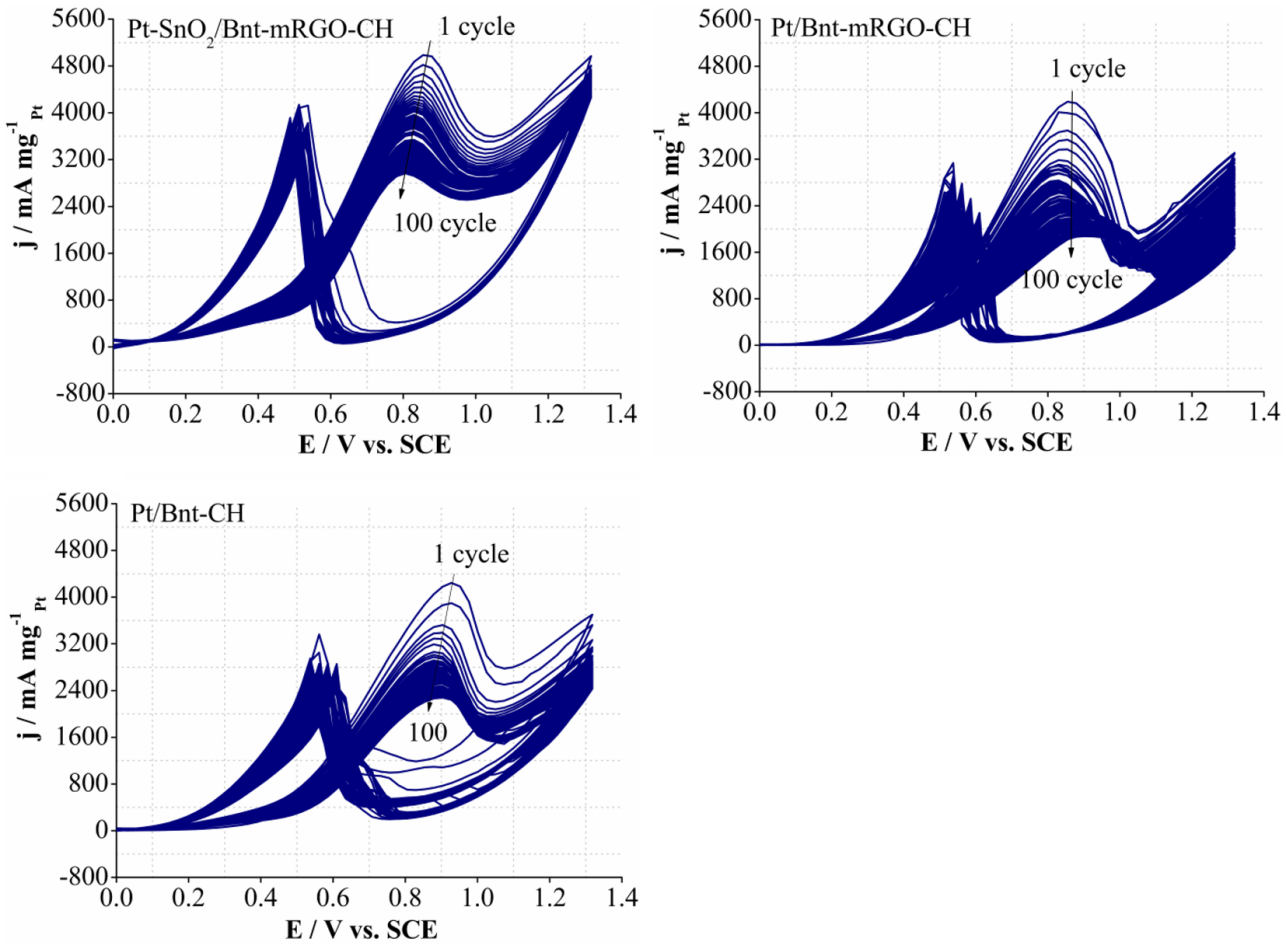
CV curves of the prepared catalysts during 100 cycles in 0.5 M H_2_SO_4_ and 0.72 M methanol solution.

The long-term stability of the synthesized catalysts for MOR was evaluated by cyclic voltammetry technique with 100 consecutive cycles (Fig. 6). The CV curves were obtained in 0.5 M sulfuric acid and 0.72 M methanol at the scan rate of 100 mV s^−1^. As shown, the anodic mass activity of MOR for all the catalysts continuously decreased during 100 cycles. This is due to the poisoning of the catalysts with the produced carbon monoxide species^73^. For the Pt-SnO_2_/Bnt-mRGO-CH catalyst, the anodic mass activity of MOR decreased by 40.52% after 100 cycles, while for Pt/Bnt-mRGO-CH and Pt/Bnt-CH catalysts, the anodic mass activity for MOR decreased by 54.02 and 46.30%, respectively after 100 cycles. Pt-SnO_2_/Bnt-mRGO-CH catalyst showed the best stability for methanol oxidation during 100 consecutive cycles, compared to other prepared catalysts.

**Figure 7:**
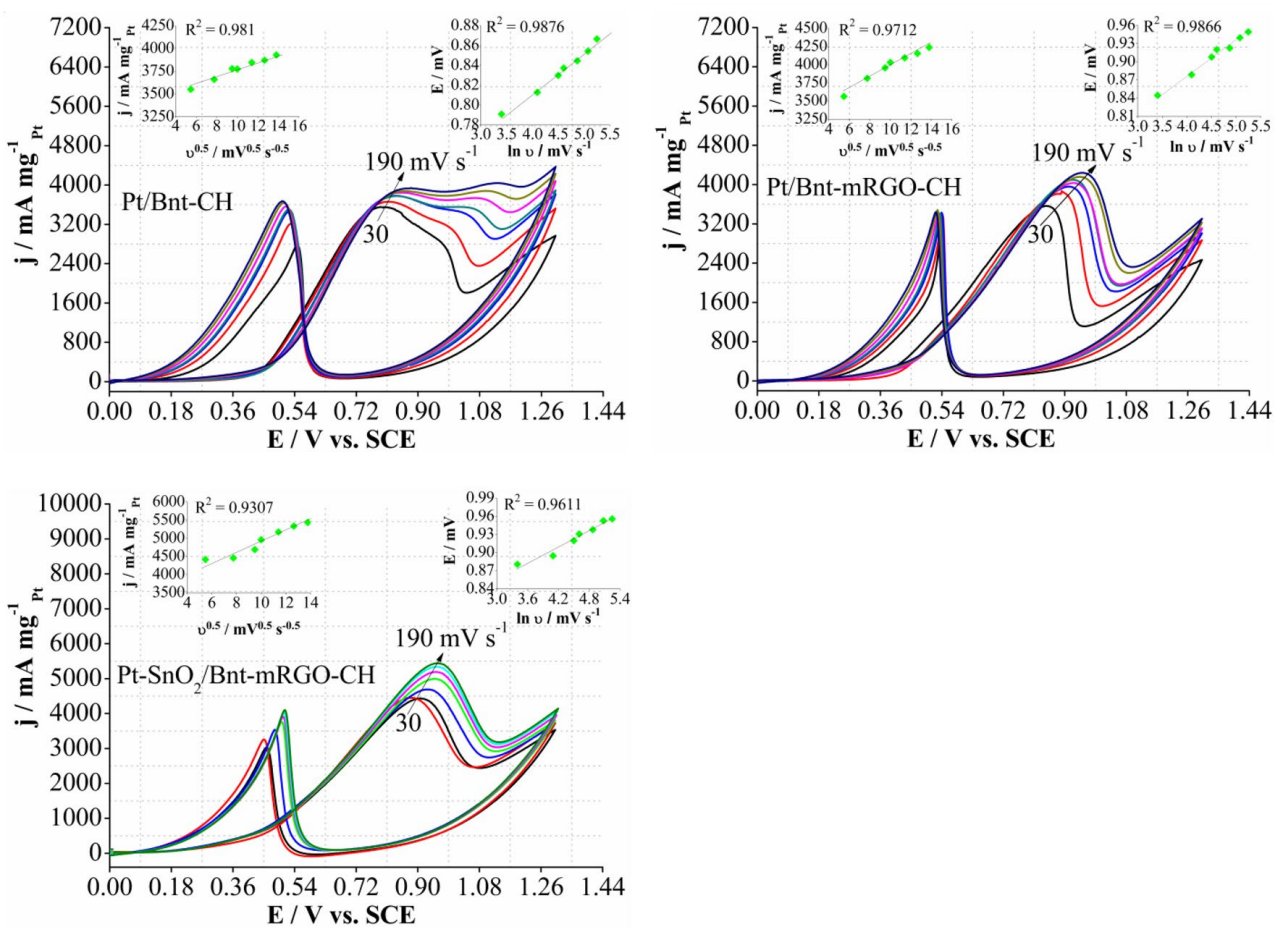
CV curves of the prepared catalysts in 0.5 M H_2_SO_4_ and 0.72 M methanol at different scan rates of 30, 60, 90, 100, 130, 160, and 190 mV s^−1^.

The catalytic performance of Pt/Bnt-CH, Pt/Bnt-mRGO-CH, and Pt-SnO_2_/Bnt-mRGO-CH catalysts were investigated for methanol electro-oxidation at different scan rates of 30, 60, 90, 100, 130, 160, and 190 mV s^−1^ in the solution containing H_2_SO_4_ 0.5 M and methanol 0.72 M (Fig. 7). As shown, the anodic peak mass activity of methanol oxidation increases with increasing the scan rate. For each catalyst, the plots of anodic peak mass activity of MOR (j) versus the square root of scan rate (ʋ^0.5^) and the plots of anodic peak potential of MOR (E) versus ln ʋ are shown in the inset of each Fig. The linear relationship between j and ʋ^0.5^ for each catalyst reveals that methanol oxidation is controlled by diffusion of methanol from the solution to the surface of electrode^74^. The linear relationship between E and ln ʋ for each catalyst shows the irreversibility of charge transfer process for methanol electro-oxidation^71^.

## Conclusions

In summary, the novel Pt-SnO_2_/Bnt-mRGO-CH nanocatalyst was synthesized, using the novel Bnt-mRGO-CH support for uniformly dispersion of nanoparticles. The as-prepared catalyst exhibited excellent catalytic activity for methanol electro-oxidation, compared to Pt/Bnt-CH and Pt/Bnt-mRGO-CH catalysts. It was found that the use of SnO_2_ nanoparticles accompanied by Pt nanoparticles and their uniform dispersion on Bnt-mRGO-CH support improved the catalytic performance of Pt catalyst for MOR by creating more available active sites for methanol electro-oxidation. Pt-SnO_2_/Bnt-mRGO-CH exhibited enhanced catalytic performance for MOR compared to Pt/Bnt-mRGO-CH and Pt/Bnt-CH nanocatalysts, due to its higher electrochemically active surface area, better stability, and higher anodic mass activity. By and large, it was demonstrated that Pt-SnO_2_/Bnt-mRGO-CH is a promising catalyst for MOR. Undoubtedly, this research is of great significance for the development of novel and efficient catalysts for methanol oxidation in direct methanol fuel cells.

## Data Availability

All data generated or analysed during this study are included in this published article.
